# Association of a high normalized protein catabolic rate and low serum albumin level with carpal tunnel syndrome in hemodialysis patients

**DOI:** 10.1097/MD.0000000000004050

**Published:** 2016-07-01

**Authors:** Wen-Hung Huang, Ching-Wei Hsu, Cheng-Hao Weng, Tzung-Hai Yen, Jui-Hsiang Lin, Meng Lee

**Affiliations:** a Department of Nephrology and Division of Clinical Toxicology, Chang Gung Memorial Hospital, Linkou Medical Center; b Chang Gung University College of Medicine; c Division of Nephrology, Department of Internal Medicine, Taoyuan General Hospital, Ministry of Health and Welfare, Taoyuan; d Graduate Institute of Clinical Medicine, Taipei Medical University; e Department of Neurology, Chang Gung Memorial Hospital, Chiayi Branch, Puzi, Taiwan, R.O.C.

**Keywords:** carpal tunnel syndrome, hemodialysis, hypoalbuminemia, inflammation, nPCR

## Abstract

Carpal tunnel syndrome (CTS) is the most common mononeuropathy in patients with end-stage renal disease (ESRD). The association between chronic inflammation and CTS in hemodialysis (HD) patients has rarely been investigated. HD patients with a high normalized protein catabolic rate (nPCR) and low serum albumin level likely have adequate nutrition and inflammation. In this study, we assume that a low serum albumin level and high nPCR is associated with CTS in HD patients. We recruited 866 maintenance hemodialysis (MHD) patients and divided them into 4 groups according to their nPCR and serum albumin levels: (1) nPCR <1.2 g/kg/d and serum albumin level <4 g/dL; (2) nPCR ≥1.2 g/kg/d and serum albumin level <4 g/dL; (3) nPCR <1.2 g/kg/d and serum albumin level ≥4 g/dL; and (4) nPCR ≥1.2 g/kg/d and serum albumin level ≥4 g/dL. After adjustment for related variables, HD duration and nPCR ≥1.2 g/kg/d and serum albumin level <4 g/dL were positively correlated with CTS. By calculating the area under the receiver-operating characteristic curve, we calculated that the nPCR and HD duration cut-off points for obtaining the most favorable Youden index were 1.29 g/kg/d and 7.5 years, respectively. Advance multivariate logistic regression analysis revealed that in MHD patients, nPCR ≥1.29 g/kg/d and serum albumin <4 g/dL, and also HD duration >7.5 years were associated with CTS. A high nPCR and low serum albumin level, which likely reflect adequate nutrition and inflammation, were associated with CTS in MHD patients.

## 1 Introduction

Carpal tunnel syndrome (CTS) is the most common mononeuropathy in patients with end-stage renal disease (ESRD), with a frequency of 8% to 31%.^[1,2]^ Long-term hemodialysis (HD) is a well-known cause of CTS.^[3]^ However, the exact causes of CTS development in patients with ESRD remain unclear. Increased plasma beta-2-microglobulin (BMG) levels in HD patients are believed to play an essential role in the pathogenesis of CTS.^[4]^ The development of dialysis amyloid depends on the duration of dialysis,^[5]^ type of membrane,^[6]^ and age of the patient.^[7]^ In addition, CTS is associated with connective tissue diseases.^[8,9]^ However, the association between chronic inflammation and CTS in HD patients has rarely been reported. Lukowsky et al^[10]^ recently studied the accuracy of using the serum albumin level and normalized protein catabolic rate (nPCR) for predicting mortality in HD patients. HD patients with a high nPCR and low serum albumin level likely have adequate nutrition and inflammation. In addition, the mortality in these patients was higher than that in patients with both high nPCR and serum albumin level. In this study, we suppose that both serum albumin level and nPCR are associated with CTS in HD patients.

## 2 Methods

This study protocol was approved by the Institutional Review Board Committee of Chang Gung Memorial Hospital. The institutional review board has waived the need for informed consent. All medical records, including medical history, laboratory data, and inclusion and exclusion criteria, were reviewed by senior nephrologists during the study period. All patient information was protected and available to only the investigators.

### 2.1 Patients

Patients were recruited from the HD centers of the Chang Gung Memorial Hospital branches in Linkou, Taipei, and Taoyuan. Only maintenance HD (MHD) patients who had undergone HD for at least 6 months and were aged ≥18 years were enrolled. Regarding hemodiafiltration (HDF), patients who had undergone HDF 3 times a week for ≥3 months were enrolled. Patients with malignancies or infectious diseases or who had been hospitalized or had undergone surgery within the previous 3 months were excluded. Diabetes mellitus was identified according to either a physician's diagnosis, antidiabetic drug treatment, or 2 subsequent analyses demonstrating fasting blood glucose levels of >126 mg/dL. Most patients underwent 4 hours of HD 3 times a week. HD was performed using single-use hollow-fiber dialyzers equipped with modified cellulose, polyamide, or polysulfone membranes. The dialysate used in all cases had a standard ionic composition with a bicarbonate-based buffer. We evaluated the prevalence of cardiovascular diseases (CVDs), including cerebrovascular disease, coronary artery disease, congestive heart failure, and peripheral vascular disease, in the patients. Hypertension was defined as the regular use of antihypertensive drugs for controlling blood pressure or at least 2 blood pressure measurements of >140/90 mm Hg. In addition, smoking behavior was analyzed. CTS diagnosis was made according to (1) signs or symptoms verified using nerve conduction examination; (2) clinical CTS diagnosis of nocturnal pain, numbness in the median nerve distribution, and a positive Tinel sign/Phalen sign; (3) prolonged sensory and/or motor latencies from the wrist to the digits innervated by the median nerve in the electrophysiological test; or (4) CTS requiring surgical release.

### 2.2 Laboratory, nutritional, and inflammatory parameters

All blood samples were obtained from the arterial end of the vascular access after the 2-day interval for HD, and were then centrifuged and stored at–80°C until use. Serum creatinine levels, nPCRs, and serum albumin levels were as nutritional markers. High-sensitivity C-reactive protein (hsCRP) levels were detected as the marker of inflammation. Serum hsCRP level was detected from immunonephelometry (Nanopia CRP; Daiichi Inc, Tokyo, Japan). Less than 0.15 mg/L was the lowest detection limit. Standard laboratory approach with automatic analyzer was used for all other biochemical parameters. The dialyzer clearance of urea, which was detected from the method by Daugirdas,^[11]^ was expressed as Kt/V_urea_. The serum calcium level was detected and corrected by serum albumin level: corrected calcium level (mg/dL) = serum calcium level + 0.8 × (4.0 − serum albumin level). Nonanuria was defined as daily urine output more than 100 mL. The nPCR of the HD patients was calculated and normalized to their body weight.^[12]^

### 2.3 Definition of hypoalbuminemia and inflammation

The most appropriate level of albumin for nutrition evaluation remains unclear. A serum albumin level of <3.6 g/dL was defined as malnutrition; this is near the lower limit of the normal range in our hospital, that is, 3.5 g/dL, and represents the 10th percentile of the definition in the Third National Health and Nutrition Examination Survey of Americans.^[13,14]^ However, according to the Kidney Disease Outcomes Quality Initiative (K/DOQI) Clinical Practice Guidelines for chronic kidney disease (CKD), a serum albumin level of ≥4.0 g/dL in MHD patients is acceptable.^[15]^ Therefore, on the basis of previous observations and guidelines, we defined hypoalbuminemia as a serum albumin level of <4.0 g/dL in MHD patients. The presence of inflammation in MHD patients was defined as an hsCRP level of >3 mg/L; this level is correlated with increased cardiovascular risk in the general population.^[16,17]^

### 2.4 Albumin and nPCR as predictors of CTS

We analyzed the association of CTS with both serum albumin level and nPCR. According to Lukowsky et al,^[10]^ patients with a high nPCR and low serum albumin likely have adequate nutrition and inflammation; patients with a low nPCR and adequate serum albumin level may have an inadequate nutritional status, but may also be less likely to have inflammation; patients with both a low nPCR and low serum albumin level may be malnourished and have inflammation; and patients with both a high nPCR and high serum albumin level are more likely to have neither of the 2 conditions. Therefore, on the basis of these assumptions and the K/DOQI Clinical Practice Guidelines,^[15]^ we divided the patients according to their nPCR and serum albumin levels into 4 groups: (1) nPCR <1.2 g/kg/d and serum albumin level <4 g/dL; (2) nPCR ≥1.2 g/kg/d and serum albumin level <4 g/dL; (3) nPCR <1.2 g/kg/d and serum albumin level ≥4 g/dL; and (4) nPCR ≥1.2 g/kg/d and serum albumin level ≥4 g/dL.

### 2.5 Statistical analysis

Data were analyzed using SPSS version 12.0 for Windows 95 (SPSS Inc, Chicago, IL). The normal distribution of variables was analyzed using the Kolmogorov–Smirnov test. A *P* value of >0.05 was considered to indicate normal distribution. Continuous variables were expressed as mean ± standard deviation and categorical variables as numbers or percentages. Chi-square or Fisher exact tests were used for analyzing the correlation among categorical variables. Comparisons between 2 groups were performed using the Mann–Whitney *U* test and Student *t* test. The data on hsCRP, intact parathyroid hormone (iPTH), and ferritin levels were log-transformed for analysis. To evaluate the variables related to CTS, multivariate logistic regression analyses in the forward method were performed to calculate the odds ratios (ORs) and 95% confidence intervals (CIs) for baseline variables, namely age; sex; body mass index (BMI); smoking status; diabetes mellitus; hypertension; previous CVD; hepatitis B virus (HBV) and hepatitis C virus (HCV) infection; HD duration; fistula for blood access; HDF; Kt/V_urea_ Daugirdes; nPCR; nonanuria status; hemoglobin (Hb) levels; serum albumin and creatinine levels; corrected calcium, inorganic phosphate, log ferritin, and log iPTH levels; hsCRP >3.0 mg/dL; and cholesterol and triglyceride levels. Discrimination was evaluated by calculating the area under the receiver-operating characteristic curve (AUROC). The cut-off point was calculated by obtaining the best Youden index (sensitivity + specificity − 1). All the nominal variables in the logistic regression were transformed into dummy coding. Missing data were removed using list-wise deletion. The level of significance was set at *P* < 0.05.

## 3 Results

### 3.1 Study population characteristics

In total, this study comprised 866 MHD patients (440 men and 426 women) with a mean MHD duration of 6.96 ± 5.35 years. Table 1 lists the patient characteristics, including age, sex, and BMI, along with biological, hematological, and HD data. Of all the patients, 50.8% were male, 22.2% had a medical history of DM, 4.7% had CVDs, 2.9% had lupus, 17.3% were habitual tobacco users, 79.6% had an AV fistula, 8.8% had CTS, 11.3% had HBV infection, and 19.4% had HCV infection. Of the 76 patients with CTS, 38 patients were diagnosed with CTS by electrophysiological test, and the other 38 patients were diagnosed with CTS by clinical examinations. In addition, 19 patients needed surgical release for CTS.

**Table 1:**
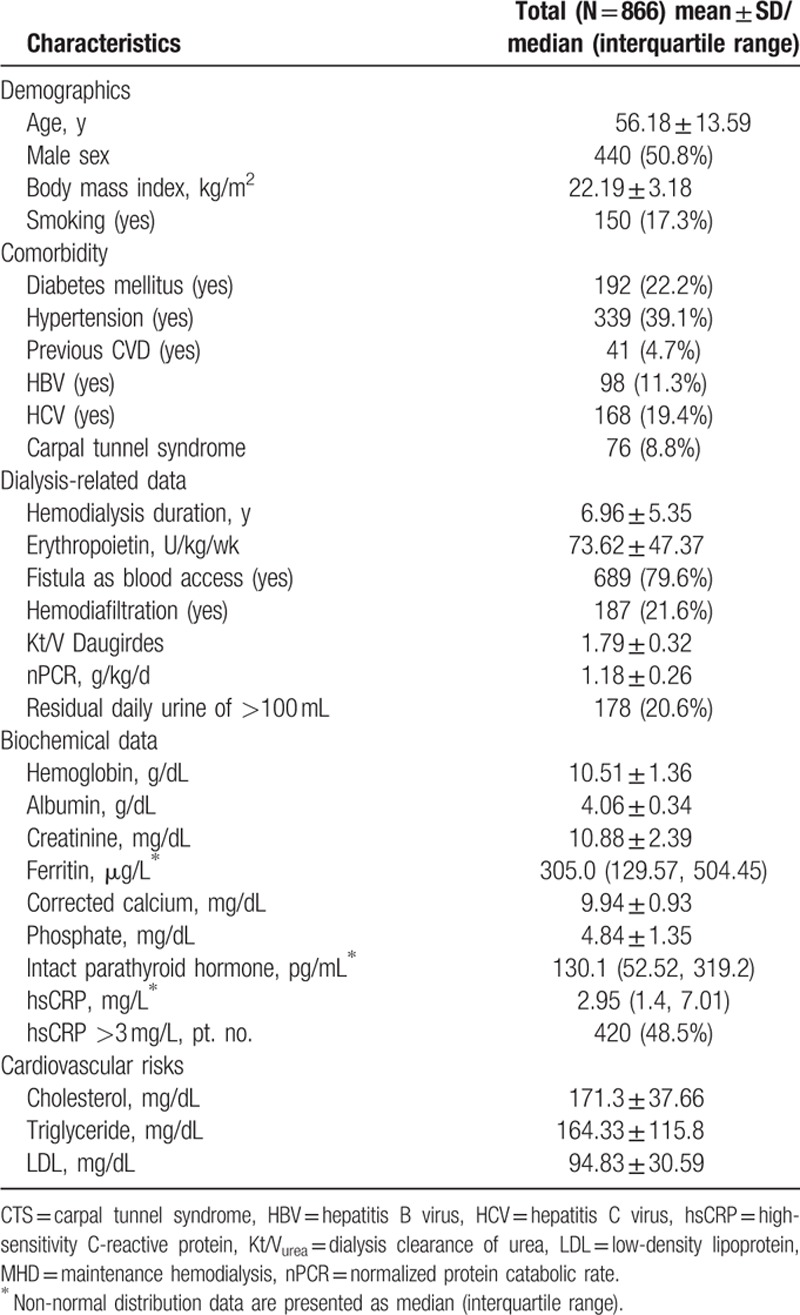
Baseline characteristics of 866 MHD patients.

Table 2 presents the subgroup analysis for patients with and without CTS. The patients with CTS had a longer HD duration (12.3 ± 5.79 vs 6.45 ± 5.02 years; *P* < 0.001), higher Hb level (10.84 ± 1.55 vs 10.48 ± 1.34 g/dL; *P* = 0.02), higher iPTH level (254.35 vs 122.25 pg/mL; *P* < 0.001), higher nPCR (1.26 ± 0.27 vs 1.18 ± 0.26 g/kg/d; *P* = 0.007), lower albumin level (3.98 ± 0.34 vs 4.07 ± 0.34 g/dL; *P* = 0.033), higher KT/V_urea_ (1.92 ± 0.36 vs 1.78 ± 0.32; *P* < 0.001), lower erythropoietin usage (60.84 ± 46.65 vs 74.85 ± 47.28 U/kg/wk; *P* = 0.014), lower DM prevalence (9.2% vs 23.4%; *P* = 0.003), higher HCV prevalence (31.6% vs 18.2%; *P* = 0.009), and lower nonanuria prevalence (7.9% vs 21.8%; *P* = 0.003).

**Table 2:**
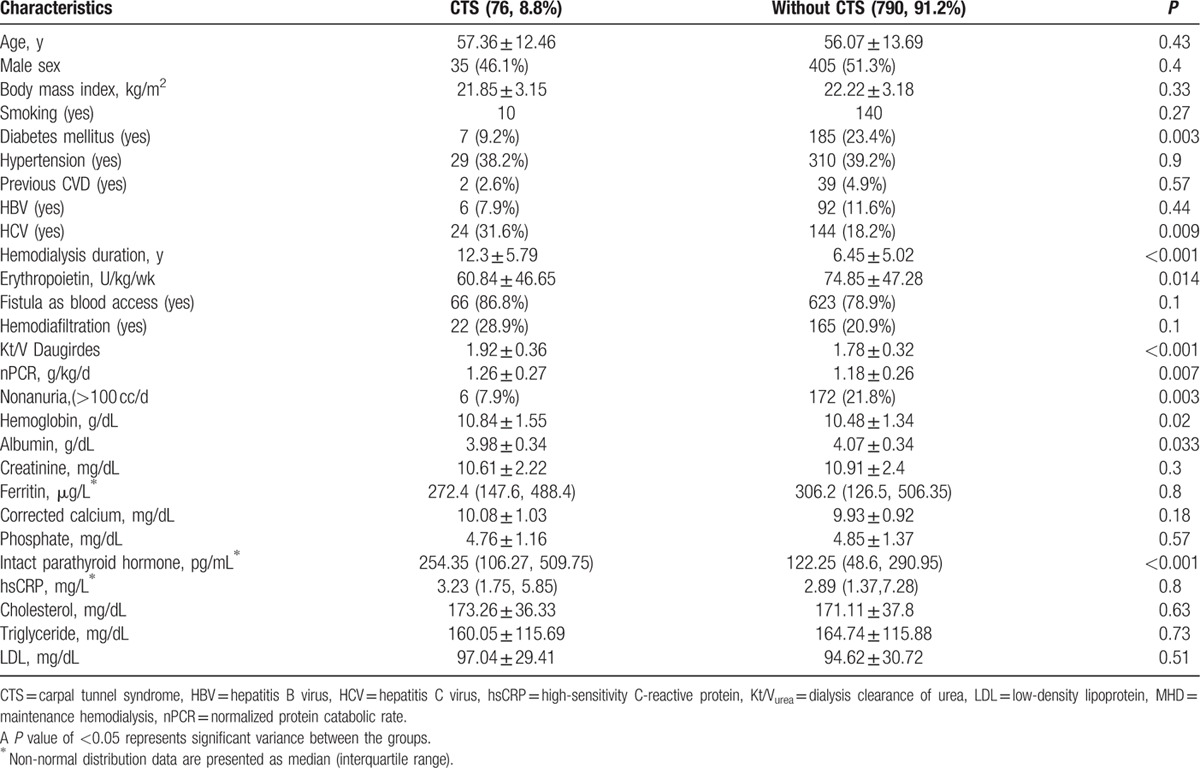
Comparison between MHD patients with CTS and without CTS.

For further investigating the influence of clinical features on CTS in HD patients, we used univariate and multivariate binary logistic regression analyses to evaluate the association between CTS and clinical variables in the patients. According to our univariate binary logistic regression results (Table 3), DM (OR 0.33, 95% CI 0.15–0.73, *P* = 0.007), HCV (OR 2.07, 95% CI 1.23–3.47, *P* = 0.006), HD duration (OR 1.17, 95% CI 1.13–1.22, *P* < 0.001), Kt/V_urea_ (OR 3.39, 95% CI 1.71–6.7, *P* < 0.001), nPCR (OR 3.17, 95% CI1.35–7.4, *P* = 0.008), nonanuria (OR 0.3, 95% CI 0.13–0.72, *P* = 0.007), Hb level (OR 1.21, 95% CI 1.02–1.43, *P* = 0.027), serum albumin level <4 g/dL (OR 2.038, 95% CI 1.26–3.27, *P* = 0.003), and log iPTH level (OR 2.62, 95% CI 1.63–4.22, *P* < 0.001) were significantly associated with CTS in the MHD patients. However, an hsCRP level of >3.0 mg/L was not associated with CTS in the MHD patients (OR 1.18, 95% CI 0.73–1.9, *P* = 0.48). Advance multivariate binary logistic regression analysis (Table 4, analysis A) revealed that after adjustment for the studied variables, nPCR (OR 3.532, 95% CI 1.436–8.691, *P* = 0.006), HD duration (OR 1.217, 95% CI 1.154–1.284, *P* < 0.001), and serum albumin level <4 g/dL (OR 1.93, 95% CI 1.137–3.278, *P* = 0.015) were significantly correlated with CTS. On the basis of the aforementioned nutrition guidelines,^[15]^ we divided the patients into 3 groups according to their nPCR: <0.8 g/kg/d, <1.2 g/kg/d, and nPCR ≥1.2 g/kg/d. The patients with nPCR ≥1.2 g/kg/d exhibited a stronger association with CTS than did those with a lower nPCR (Table 4, analysis B). Compared with patients with nPCR ≥1.2 g/kg/d and serum albumin level ≥4 g/dL, those with nPCR ≥1.2 g/kg/d and serum albumin level <4 g/dL had a stronger positive correlation with CTS (OR 2.452, 95% CI 1.22–4.92, *P* = 0.012; Table 4, analysis C).

**Table 3:**
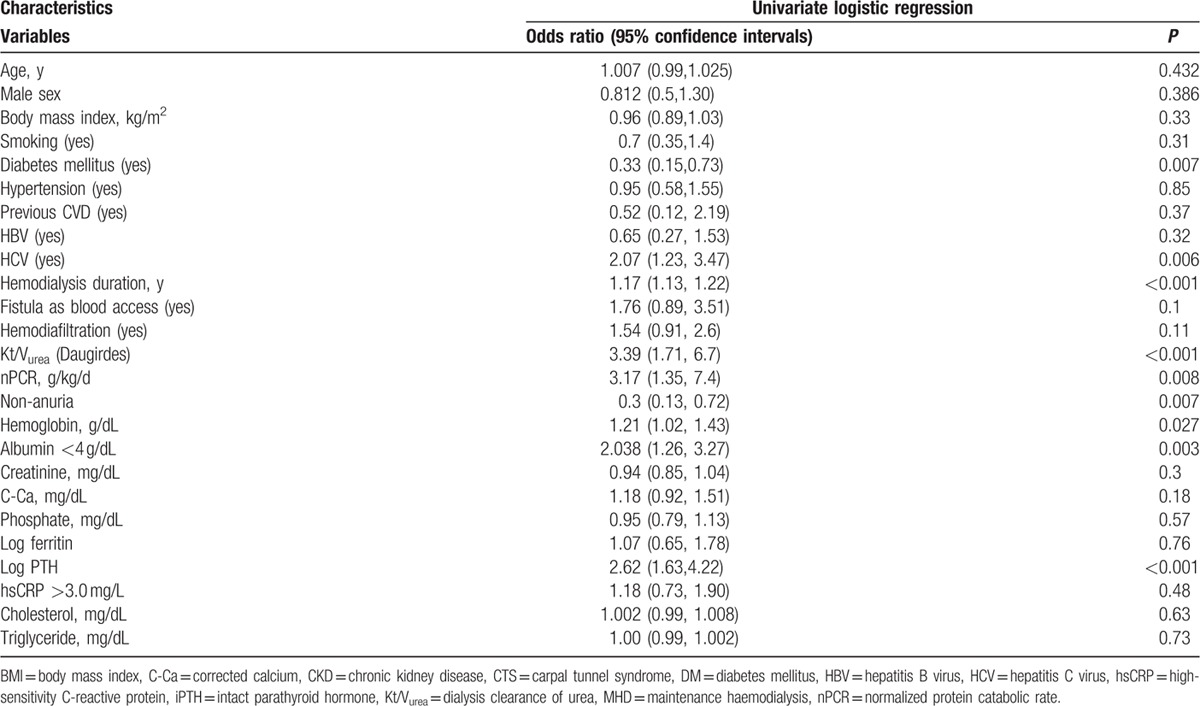
Univariate logistic regression analysis between CTS and clinical variables in MHD patients.

**Table 4:**
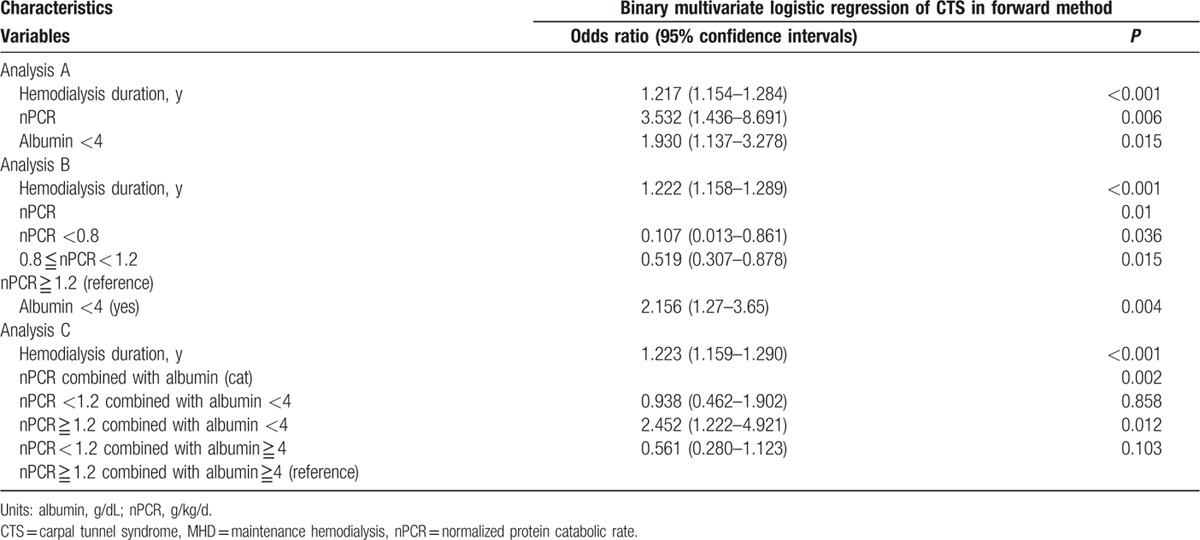
Multivariate logistic regression analysis (forward method) between CTS and clinical variables in MHD patients.

### 3.2 AUROC for NPCR and HD duration

**Figure 1:**
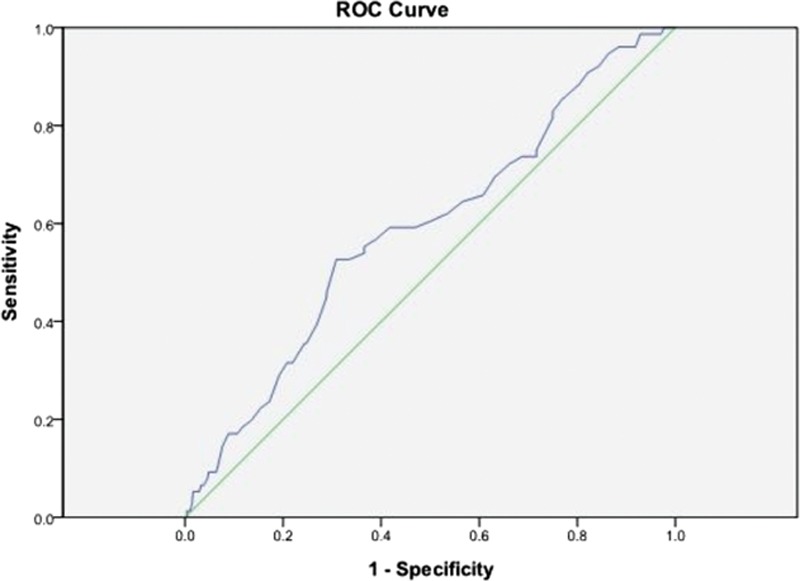
Computation for the AUROC confirmed favorable discriminatory power of nPCR (AUROC = 0.588 ± 0.035, 95% CI 0.52–0.65, *P* *=* 0.011). The cut-off point calculated by obtaining the best Youden index was 1.29 g/kg/d, with a sensitivity of 53% and specificity of 69%. AUROC = area under the receiver-operating characteristic curve, CI = confidence interval.

**Figure 2:**
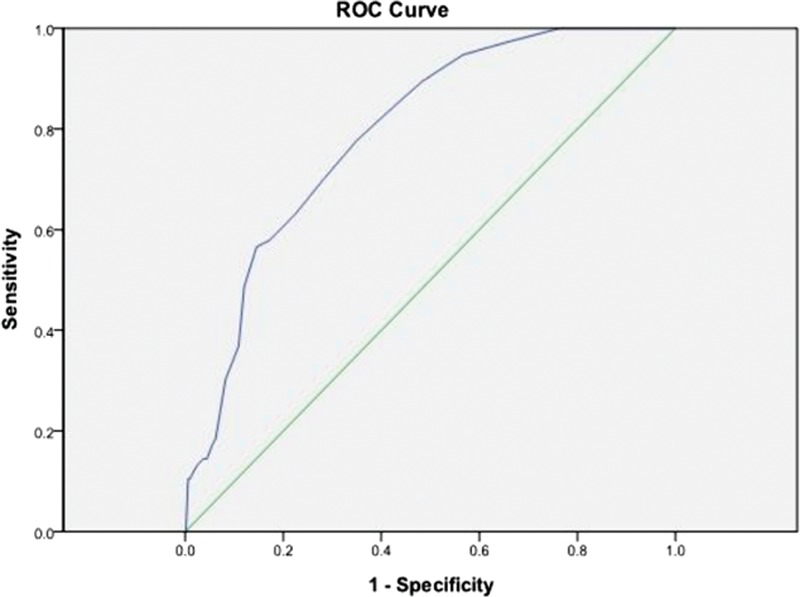
The cut-off point calculated by obtaining the best Youden index of the HD duration was 7.5 years (AUROC = 0.792 ± 0.023, 95% CI 0.74–0.83, *P* < 0.001), with a sensitivity of 78% and specificity of 66%. AUROC = area under the receiver-operating characteristic curve, CI = confidence interval, HD = hemodialysis.

A higher nPCR was associated with CTS. Computation for the AUROC confirmed favorable discriminatory power of nPCR (AUROC = 0.588 ± 0.035, 95% CI 0.52–0.65, *P* *=* 0.011; Fig. 1). The cut-off point calculated by obtaining the best Youden index was 1.29 g/kg/d, with a sensitivity of 53% and specificity of 69%. Similarly, the cut-off point calculated by obtaining the best Youden index of the HD duration was 7.5 years (AUROC = 0.792 ± 0.023, 95% CI 0.74–0.83, *P* < 0.001; Fig. 2), with a sensitivity of 78% and specificity of 66%. Accordingly, HD duration ≥7.5 years and nPCR ≥1.29 were used in advance multivariate binary logistic regression analysis for evaluating their association with CTS (Table 5). HD duration ≥7.5 years (OR 5.2, 95% CI 2.94–9.22, *P* < 0.001), nPCR ≥1.29 g/kg/d (OR 2.42, 95% CI 1.45–4.03, *P* = 0.001), and serum albumin level <4 g/dL (OR 2.03, 95% CI 1.21–3.41, *P* = 0.007) were associated with CTS (Table 5, analysis D). In MHD patients, nPCR ≥1.29 g/kg/d and serum albumin level <4 g/dL (OR 2.74, 95% CI 1.45–5.17, *P* = 0.002) were associated with CTS (Table 5, analysis E). To further investigate the association of high nPCR (nPCR ≥1.29 g/kg/d) and hypoalbuminemia (serum albumin level <4 g/dL) with CTS prevalence, we categorized high and low nPCRs and albumin levels, and analyzed the results by using the Fisher exact test. Table 6 shows that patients with nPCR ≥1.29 g/kg/d and serum albumin level <4 g/dL had the highest CTS prevalence (22.1%). In addition, we observed that HCV infection exhibited a mild positive association with CTS (*r* = 0.096, *P* = 0.005). However, after adjustment for HD duration, this positive correlation was not observed (*r* = −0.059, *P* = 0.084).

**Table 5:**
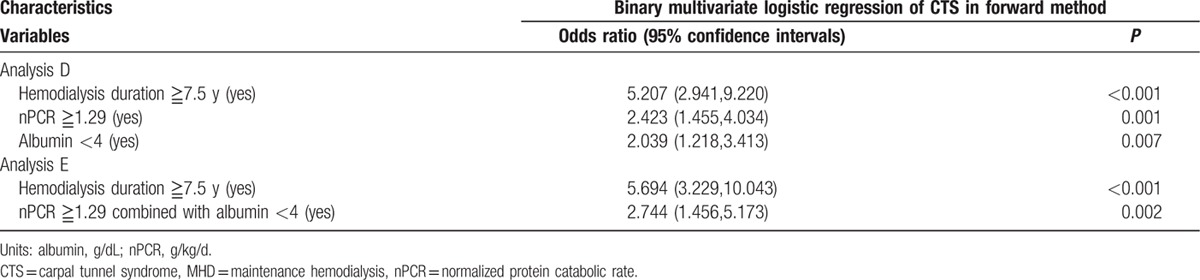
Multivariate logistic regression analysis (forward method) between CTS and clinical variables in MHD patients.

**Table 6:**
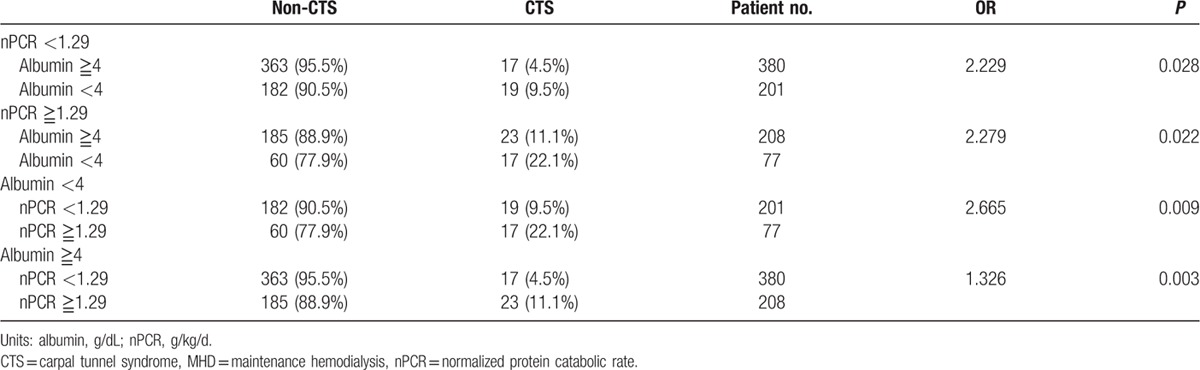
Fisher exact test of incidence of CTS with degrees by nPCR and serum albumin.

### 3.3 Analysis of Albumin, hsCRP, and NPCR

**Figure 3:**
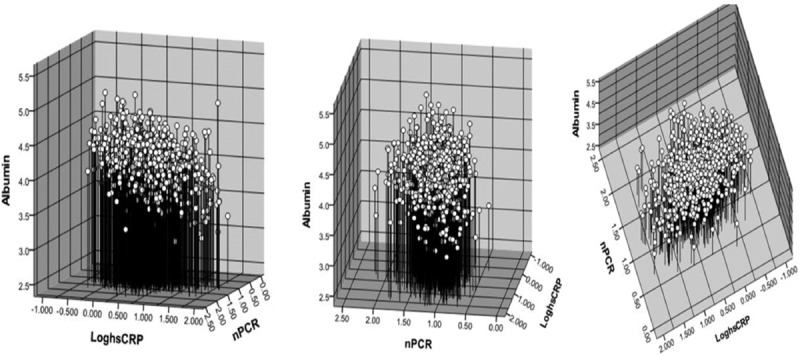
Relationship between the dependent variable, albumin and nPCR, and log-transformed hsCRP concentration is represented in 3-dimensional style. The following formula was the result for determining the correlation between serum albumin level and protein intake and inflammation: albumin = 3.95 − (0.193 × log hsCRP) + (0.181 × nPCR). The relationship between variables: albumin and log hsCRP: *r* = −0.304, *P* < 0.001; albumin and nPCR: *r* = 0.174, *P* < 0.001; log hsCRP and nPCR: *r* = −0.102, *P* = 0.003. hsCRP = high-sensitivity C-reactive protein, nPCR = normalized protein catabolic rate.

In this cross-sectional study, we observed an inverse correlation between log hsCRP and serum albumin level (*r* = −0.3, *P* < 0.001) and a direct correlation between nPCR and serum albumin level (*r* = 0.174, *P* < 0.001). In addition, an inverse correlation was observed between log hsCRP and nPCR (*r* = −0.1, *P* = 0.003). Figure 3 shows the effects of dietary protein intake and inflammation on the serum albumin level in 3-dimensional style. The following formula was the result for determining the correlation between serum albumin level and protein intake and inflammation: albumin = 3.95 − (0.193 × log hsCRP) +  (0.181 × nPCR).

## 4 Discussion

A low serum albumin level in HD patients is associated with both malnutrition and inflammation,^[18,19]^ and nPCR is associated with dietary protein intake.^[20]^ Albumin and nPCR were determined as the independent predictors of mortality in MHD patients.^[21,22]^ In this study, we evaluated the effects of these predictors by dividing MHD patients into 4 groups according to their nPCR and serum albumin level, and then examined CTS prevalence in these patients. The patients with a high nPCR and low serum albumin level had the highest CTS prevalence (22.1%).

Hemodialysis duration is associated with CTS prevalence, and the frequency of CTS in HD patients is approximately 8% to 31%. A study evaluating long-term HD found that CTS developed in 50% of patients after a mean HD duration of 11.1 years.^[23]^ In our study, the mean HD duration was approximately 6.96 years, which is shorter that that reported in other studies; thus, it was not surprising that the CTS prevalence in the current HD patients was approximately 8.8%.

Kopeć et al^[3]^ reported a positive correlation between HCV infection and CTS in HD patients. They reported that the longer patients are dialyzed, the higher their probabilities of HCV infection. In our study, patients with HCV infection had a higher frequency of CTS. However, after adjustment for HD duration by using multivariate logistic regression or Pearson correlation, this association was not observed. In our study, patients with HCV infection had a longer HD duration (12 ± 6.7 vs 5.7 ± 4.1 years). In our HD center, the rate of HCV infection in the previous decade was 0; it was also lower in the previous 10 to 20 years, during which the rate ranged from 0.17% to 0.28%. Thus, in the current study, we observed the effect of HD duration on CTS rather than the effect of HCV infection itself.

Plasma BMG level is believed to play a crucial role in the pathogenesis of dialysis-related amyloidosis and CTS in HD patients. However, the role of inflammation in the pathogenesis of dialysis-related CTS has rarely been reported. Theaker et al reported that amyloid is generally considered to be an inert substance causing a mild inflammatory reaction. In addition, he reported a physical association between the amyloid deposits and foci of granulomatous inflammation.^[24]^ Thus, we know that inflammation plays a crucial role^[25]^ in CTS, and we could view dialysis-related CTS as a condition of chronic inflammation. Inflammation is strongly associated with increased cardiovascular risk in patients with renal failure.^[26–28]^ CRP is a crucial predictor of all-cause and cardiovascular mortality in HD patients.^[27]^ In our study, CTS prevalence was not associated with hsCRP >3.0 mg/L or any hsCRP level. Moreover, after including log hsCRP level as a continuous factor in multivariate logistic regression, log hsCRP level was still not associated with CTS (OR 1.008, *P* = 0.97). This result is consistent with that reported by Curatola et al.^[29]^ Possible reasons why log hsCRP level was not associated with CTS are as follows: (1) the lower sensitivity limit of the hsCRP assay used in this study may have masked clinically significant levels of inflammation; (2) the CRP values may have limited the detection of inflammation in the HD patients; (3) low hsCRP levels were observed in our patients (median hsCRP: 2.95 mg/L); and (4) CRP may not be suitable for testing chronic inflammation including granulation.

In HD patients, though their nPCR is high, serum albumin levels decrease because of progressive inflammation.^[30]^ Although this may reflect a mismatch between nitrogen consumption and catabolism, albumin synthesis is suppressed during periods of inflammation,^[31]^ and the fractional rate of albumin catabolism is greater than that observed normally^[32]^ for a given serum albumin level.^[33]^ This suggests that albumin levels are strongly influenced by the effects of inflammation, regardless of nitrogen availability. In addition, nPCR is associated with dietary protein intake,^[20]^ and K/DOQI Clinical Practice Guidelines recommend a daily protein intake of 0.6 to 0.8 g/kg/d for CKD patients and 1.2 and 1.3 g/kg/d for MHD patients, which may contribute to low nPCR levels at baseline.^[34]^ The effects of nPCR >1.3 g/kg/d in patients with CKD remain unclear. A rapid increase in nPCR or nPCR >1.4 g/kg/d may result in a negative nitrogen balance, increase in catabolic rate during infection, or inflammation. The protective effect of an increase in nPCR on mortality is certain for only the first 6 to 9 months of HD.^[20]^ Similarly, in our study, we observed that nPCR ≥1.29 g/kg/d was the optimal value for predicting the association of CTS in the AUROC analysis. Lukowsky et al^[10]^ recently reported that nPCR ≥1 g/kg/d and serum albumin level <3.5 g/dL independently predicted mortality in HD patients. Consistent with the results of previous studies,^[10]^ we observed that after adjustment for vintage, nPCR ≥1.29 g/kg/d and serum albumin level <4 g/dL were independently and positively correlated with CTS in HD patients.

To our knowledge, evaluating the association of nPCR and serum albumin levels with CTS prevalence is limited. Our study was novel in that it examined the effects of serum albumin level and nPCR by separating the effects of malnutrition and inflammation associated with low albumin levels. However, this study has some limitations. First, it was adopted a single-center/cross-sectional study design. However, because of the single-center study design, the nutritional health education of the patients was relatively consistent. Second, our study focused on inflammation; however, apart from hsCRP, we did not measure any other inflammation markers such as α1-antitrypsin, α2-macroblobulin, and α1-acid glycoprotein. In our future work, we plan to evaluate the role of these inflammation markers. Third, we did not have access to information on the β2-microglobulin levels of the patients, which may have potentially helped us in clarifying the correlation between amyloidosis and inflammation.

## 5 Conclusions

In this cross-sectional study, we observed that a high nPCR and low serum albumin level is associated with CTS in MHD patients. Additional studies are required to clarify the role of inflammation and malnutrition in the pathogenesis of CTS in MHD patients.
